# Ischemic Colitis with Perforation Occurring 15 Months after Colorectal Cancer Surgery in a Patient with Severe Heart Failure: A Case Report

**DOI:** 10.70352/scrj.cr.25-0566

**Published:** 2025-12-23

**Authors:** Ryosuke Machi, Daisuke Yamamoto, Haruka Kubo, Shunsuke Takenaka, Hiroyuki Tanaka, Kazuyoshi Mitta, Hiroshi Saito, Kenta Doden, Yusuke Sakimura, Kengo Hayashi, Saki Hayashi, Ryota Matsui, Hiroto Saito, Toshikatsu Tsuji, Hideki Moriyama, Jun Kinoshita, Noriyuki Inaki

**Affiliations:** Department of Gastrointestinal Surgery, Kanazawa University Hospital, Kanazawa, Ishikawa, Japan

**Keywords:** ischemic colitis, colorectal cancer, postoperative complications, colonic perforation, heart failure

## Abstract

**INTRODUCTION:**

Ischemic colitis is a common gastrointestinal disorder. However, severe cases requiring surgical intervention are rare. Severe ischemic colitis is particularly uncommon following surgery for colorectal cancer, with a reported incidence of only 0.7%. To our knowledge, there have been no previous reports of ischemic colitis leading to perforation in the late postoperative period following colorectal cancer surgery. Herein, we report a rare case of ischemic colitis that developed following surgery for transverse colon cancer and progressed to colonic perforation despite conservative treatment, ultimately necessitating emergency surgery.

**CASE PRESENTATION:**

A 60-year-old man with a history of dilated cardiomyopathy, chronic kidney disease, and stage III colorectal cancer underwent laparoscopic anterior resection 15 months prior. The patient presented with lower abdominal pain, hematochezia, and fever. CT and colonoscopy revealed extensive ischemic changes from the descending to the sigmoid colon, leading to a diagnosis of ischemic colitis. The patient’s condition failed to improve despite conservative treatment, and he subsequently developed cerebral infarction and worsening heart failure during hospitalization. In the 6th week of admission, he experienced perforative peritonitis, necessitating high-risk emergency surgery consisting of left hemicolectomy, rectal resection, and transverse colostomy. Pathological examination revealed no malignancy, consistent with the ischemic changes. The patient gradually recovered under intensive care and was discharged on POD 44, after 86 days of hospitalization.

**CONCLUSIONS:**

This case highlights the challenges in managing ischemic colitis in patients with severe heart failure after colorectal surgery. Vigilant monitoring and early surgical intervention are crucial for high-risk individuals.

## Abbreviations

AcMCA
: accessory middle colic artery
CAPOX
: capecitabine and oxaliplatin
CRP
: C-reactive protein
IMA
: inferior mesenteric artery
IMV
: inferior mesenteric vein
Lt-MCA
: left branch of the middle colic artery
SMA
: superior mesenteric artery
UICC
: Union for International Cancer Control

## INTRODUCTION

Ischemic colitis is an acute intestinal ischemic disease characterized by the sudden onset of abdominal pain and hematochezia. It accounts for approximately 16.0%–19.3% cases of acute lower gastrointestinal bleeding, and is a common cause of acute abdomen encountered in clinical practice.^1–3)^ Its pathophysiology is multifactorial, involving both vascular and intestinal factors. Although patients who have undergone surgery for colorectal cancer possess both types of risk factors, the incidence of ischemic colitis in this population remains rare, reported at only 0.7%.^4)^ Moreover, to our knowledge, there have been no previous reports of ischemic colitis leading to perforation in the late postoperative period following colorectal cancer surgery.

Herein, we describe a rare case of ischemic colitis that developed 15 months after colorectal surgery in a patient with dilated cardiomyopathy and eventually progressed to intestinal perforation. This report underscores the importance of multidisciplinary management of such complex cases.

## CASE PRESENTATION

The patient, a 60-year-old man, had a history of diabetes mellitus, hypertension, deep vein thrombosis of the right lower limb, and chronic heart failure due to dilated cardiomyopathy. Left ventricular ejection fraction was approximately 20%. In June 2023, the patient presented with worsening epigastric pain. CT revealed a mass with luminal narrowing at the splenic flexure of the colon. An endoscopic colonic stent was placed under suspicion of obstructive colon cancer. A biopsy of the stenotic lesion confirmed adenocarcinoma. Owing to severely impaired cardiac function, perioperative management was considered extremely difficult, and the patient was referred to our institution for further evaluation and treatment.

**Fig. 1:**
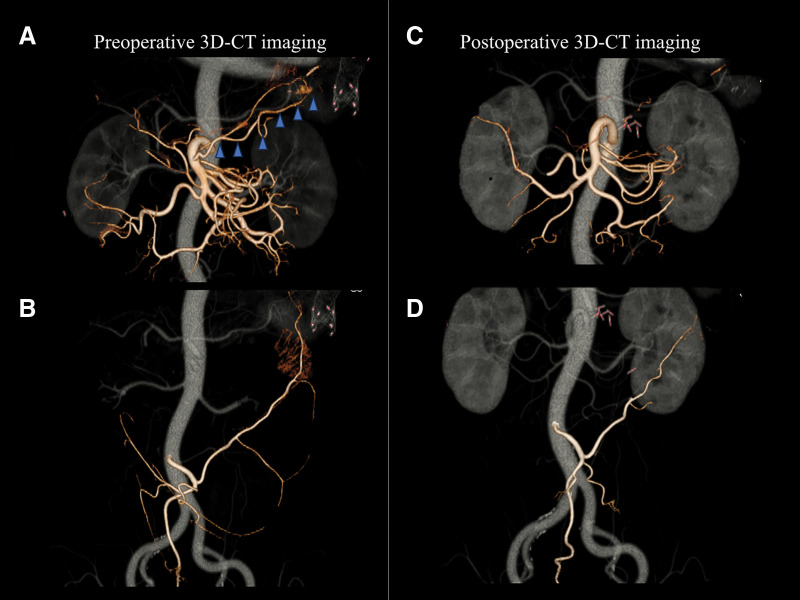
3D CT image reconstructed from contrast-enhanced CT. (**A**) Preoperative imaging revealed SMA-derived blood flow, with the AcMCA (arrowhead) serving as the main feeder to the tumor. (**B**) Preoperative imaging confirmed maintained perfusion from the IMA. (**C**) Postoperative imaging showing that blood flow from the AcMCA and Lt-MCA is no longer present. (**D**) IMA perfusion unchanged after surgery. AcMCA, accessory middle colic artery; IMA, inferior mesenteric artery; Lt-MCA, left branch of the middle colic artery; SMA, superior mesenteric artery

At our hospital, a detailed workup led to the diagnosis of transverse colon cancer (cT4aN1bM0, cStage IIIB), based on the 8th edition of the UICC TNM classification.^5)^ Cardiac evaluation revealed that surgery was feasible with intensive perioperative care in the ICU. The patient underwent a laparoscopic partial colectomy with D3 lymphadenectomy and functional end-to-end anastomosis. The tumor was primarily supplied by the AcMCA (**Fig. 1A**), which, together with its accompanying vein, bifurcated at the inferior border of the pancreas. The Lt-MCA and IMV were also divided. The operation lasted 3 h 49 min with minimal blood loss (30 g).

Pathological examination revealed a moderately-to-poorly differentiated tubular adenocarcinoma. The final pathological stage was pT4a(SE)N2a and pStage IIIC based on the 8th edition of UICC TNM classification.^5)^ The tumor exhibited mismatch repair proficiency and harbored wild-type *RAS* and *BRAF* mutations. The postoperative recovery was uneventful, and the patient was discharged on POD 8. The patient subsequently completed 8 cycles of adjuvant CAPOX chemotherapy, starting in October 2023.

Shortly thereafter, the patient developed right-sided hemiparesis and dysarthria, leading to a diagnosis of cerebral infarction. Atrial fibrillation was also identified at that time, and anticoagulation therapy was initiated. Subsequently, treatment for pneumonia was also administered. Routine surveillance colonoscopy was not possible under this course.

**Fig. 2:**
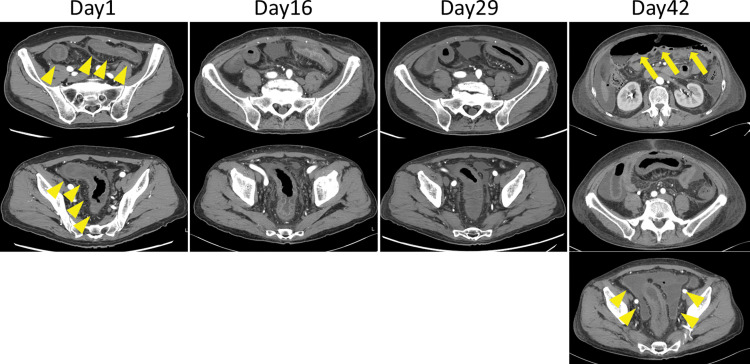
Abdominal CT findings. Day 1: Circumferential wall thickening from the descending colon to the upper rectum (arrowheads). Days 16, 29: No clear improvement is observed. Day 42: The contrast enhancement of the sigmoid colon is weak with free intraperitoneal air (arrows) and ascites (arrowheads).

**Fig. 3:**
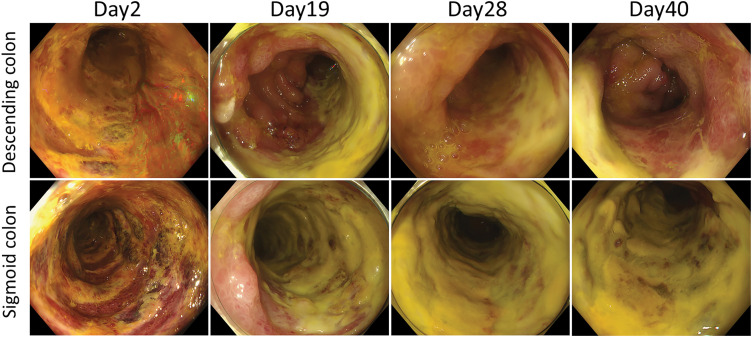
Colonoscopy findings. Day 2: Extensive ischemic changes from the descending to the sigmoid colon with near-complete mucosal loss in the sigmoid colon. Day 19: Ischemic changes appear improved; however, widespread deposits are suspected to comprise a white coating on the mucosa. Days 28, 40: No clear improvement is observed.

In December 2024, the patient presented with lower abdominal pain, hematochezia, and fever. CT revealed circumferential wall thickening extending from the descending colon to the upper rectum (**Fig. 2**). Colonoscopy showed extensive circumferential ischemic changes from the descending to the sigmoid colon, with near-complete loss of normal mucosa in portions of the sigmoid colon (**Fig. 3**). Biopsy findings were consistent with ischemic inflammation, and stool cultures tested negative for pathogenic organisms. We diagnosed ischemic colitis and commenced conservative management, including bowel rest, intravenous fluids, and antibiotics.

**Fig. 4:**
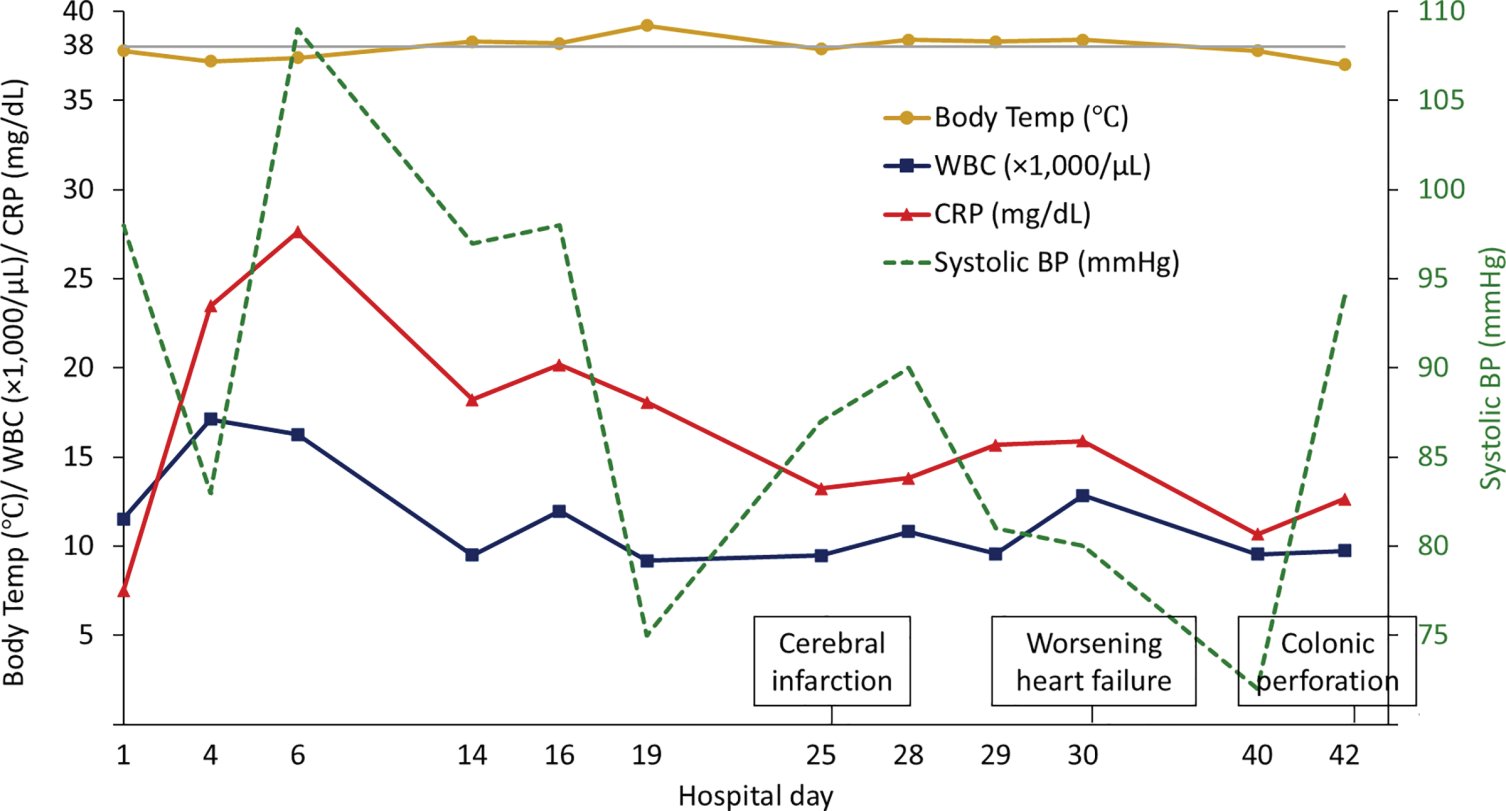
In-hospital clinical course. Body temperature, WBC count, serum CRP levels, and systolic blood pressure over time in the patient. BP, blood pressure; CRP, C-reactive protein; WBC, white blood cell

The patient exhibited persistent fever and markedly elevated CRP levels, reaching a peak of 27 mg/dL (**Fig. 4**). As hematochezia, abdominal pain, and inflammatory markers on blood tests gradually improved, he requested to resume oral intake and expressed a desire to walk outside around the 2nd week of hospitalization. However, no clear improvement was observed on CT or endoscopic examinations (**Figs. 2** and **3**). After discussing the treatment plan for ischemic colitis with gastroenterologists, it was decided that inpatient conservative management should continue.

On hospitalization day 25, the patient developed right upper limb weakness and was diagnosed with cerebral infarction. On day 30, the patient experienced a tachyarrhythmic episode due to atrial fibrillation, which worsened heart failure and required intervention by the cardiology team. The patient was initiated on an angiotensin receptor-neprilysin inhibitor, in addition to diuretics and β-blockers, which led to further hypotension.

On day 40, the abdominal pain worsened, and CT showed free intraperitoneal air, indicating acute diffuse peritonitis due to colonic perforation (**Fig. 2**). Although emergency surgery was deemed necessary, the severely impaired cardiac function of the patient posed an extremely high operative risk. The surgical risk was evaluated in consultation with anesthesiologists. After providing a thorough explanation and obtaining informed consent, we decided to proceed with emergency surgery.

**Fig. 5:**
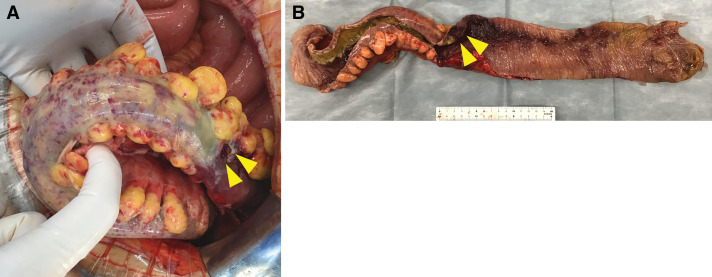
Intraoperative findings and resected specimen. (**A**) View during emergency laparotomy. (**B**) Photograph showing the surgically resected left colon and the upper rectum. The yellow arrowheads indicate the perforation of the sigmoid colon.

When the abdomen was opened, fecal contamination was observed in the peritoneal cavity. The colon, from the rectum to the splenic flexure, was discolored from black to purple, with the most severe inflammation and perforation noted in the sigmoid colon. Segmental resection of the left colon and upper rectum was performed, followed by transverse colostomy (**Fig. 5**). The operation lasted 2 h and 12 min, with 80 g of blood loss. Histopathological examination revealed no evidence of malignancy or specific pathological features, consistent with ischemic changes.

The patient was transferred from the ICU on POD 4. Weaning off dobutamine, a vasoactive agent, was managed in collaboration with cardiologists and was completed on day 20 based on the judgment of the cardiologists. The patient was discharged on POD 44, after 86 days of hospitalization.

## DISCUSSION

Ischemic colitis is the most common form of intestinal ischemia, with an incidence of 4.5–44 cases per 100000 population.^6)^ Its pathogenesis is multifactorial and involves vascular factors such as arterial stenosis, decreased mesenteric blood flow, and microthrombosis, as well as intestinal factors, such as increased intraluminal pressure and bacterial involvement.^7)^ In most cases, ischemic colitis resolves with conservative treatment; however, in rare instances, it can progress to a severe form requiring surgical intervention.^8)^

Postoperative ischemic colitis following colorectal cancer surgery is relatively uncommon, with an incidence of approximately 0.7%.^4)^ The absence of blood flow from the SMA system and venous congestion due to division of the AcMCA, Lt-MCA (**Fig. 1C**), and IMV during the initial surgery might have contributed to the ischemic injury in this case. However, the 15-month interval between surgery and the onset of ischemic colitis indicates that additional factors beyond the surgical procedure may have been involved. Although blood flow from the IMA remained unchanged before and after surgery (**Fig. 1B** and **1D**), the loss of SMA-derived perfusion may have heightened susceptibility to ischemic colitis, particularly in the context of reduced cardiac output.

Although chemotherapy-related ischemic colitis is rare, there have been reports of association with specific agents, particularly taxanes.^9–11)^ Our patient received adjuvant CAPOX therapy. While a few reports^12,13)^ have suggested an association between capecitabine and ischemic colitis, these cases developed during active chemotherapy. Our patient developed ischemic colitis 8 months after completing CAPOX, making a direct causal relationship uncertain.

The presence of cardiac and neurological comorbidities was significant in the pathophysiology of this case. Previous large-scale studies have shown that 14.3% of patients with ischemic colitis had underlying heart failure and that those with heart failure had significantly higher rates of surgical intervention and mortality.^14)^ This patient was already in a state of systemic hypoperfusion due to severe heart failure, which likely contributed to the development of cerebral infarction. The ischemic colitis that subsequently developed was further exacerbated by episodes of tachyarrhythmia due to atrial fibrillation and worsening heart failure, leading to impaired intestinal perfusion and eventually resulting in colonic perforation.

The clinical course of this case was atypical. The patient had persistent fever and elevated CRP, but the hematochezia, abdominal pain, and inflammatory markers gradually improved. By the 2nd week of hospitalization, he appeared to be recovering clinically. In ischemic colitis, the colonic mucosa typically regenerates quickly, with clinical improvement often observed within 2–3 days.^15)^ In fact, 88.4% of patients improve with conservative treatment within 1 month, and only 0.36% require surgical intervention.^16)^ At the 2-week point, after discussion with gastroenterologists, we decided to continue conservative management, prioritizing the clinical improvement of the patient and considering the high surgical risk.

However, in retrospect, more weight should have been given to the absence of improvement on serial CT and endoscopic findings. During the 3rd to 4th weeks of hospitalization, the patient developed cerebral infarction and worsening heart failure, resulting in the loss of a surgical window for a planned procedure. Ultimately, the condition progressed to colonic perforation, the worst possible outcome.

Given this progression, we now believe that earlier consideration of less invasive surgical options such as diverting stoma creation should have been made at the 2-week mark. Furthermore, the timing of surgery should have been discussed from an earlier stage through a multidisciplinary approach involving anesthesiologists and cardiologists to comprehensively assess the surgical feasibility and perioperative risks. This case highlights that surgical decision-making must strike a delicate balance between premature intervention and delayed treatment. Individualized treatment strategies, developed through multidisciplinary discussions, are crucial.

## CONCLUSIONS

This rare case of perforated ischemic colitis occurring 15 months following colorectal cancer surgery in a patient with severe heart failure underscores the challenges in managing such complications. Early recognition, multidisciplinary decision-making, and timely surgical intervention are essential for improving outcomes in high-risk patients.

## DECLARATIONS

### Availability of data and materials

The datasets supporting the conclusions of this article are included within the article.
